# Combination Immunotherapy and Yttrium-90 Radioembolization in Hepatocellular Carcinoma: Biological Rationale, Clinical Evidence, and Future Directions

**DOI:** 10.3390/cancers18111817

**Published:** 2026-06-01

**Authors:** Edward Wolfgang Lee, Ravneet Nagra

**Affiliations:** 1 Interventional Radiology, Department of Radiology, UCLA Medical Center, 757 Westwood Plaza, Suite 2125, Los Angeles, CA 90095, USA; 2 Liver Transplant Surgery, Department of Surgery, UCLA Medical Center, 757 Westwood Plaza, Suite 2125, Los Angeles, CA 90095, USA

**Keywords:** hepatocellular carcinoma, Y-90 radioembolization, immunotherapy, interventional radiology, checkpoint inhibitors

## Abstract

**Background/Objectives**: The integration of locoregional and systemic therapies represents a promising strategy in hepatocellular carcinoma (HCC). Yttrium-90 (Y-90) radioembolization provides durable local tumor control, while immune checkpoint inhibitors (ICIs) improve systemic disease outcomes. This review evaluates the biological rationale, clinical evidence, and emerging role of combination Y-90 radioembolization and immunotherapy in HCC. **Methods**: A semi-systematic (PRISMA-informed) literature review of PubMed/MEDLINE through September 2025 was conducted, including clinical trials, retrospective and prospective studies, and translational investigations evaluating Y-90 radioembolization, immunotherapy, and their combination. **Results**: Preclinical and translational studies demonstrate that Y-90 radioembolization induces immunogenic cell death, enhances antigen presentation, and activates immune pathways including interferon signaling and STING-mediated responses, supporting a mechanistic basis for potential synergy with ICIs. Early clinical studies, including phase I/II trials, report objective response rates ranging from approximately 30% to 41.5% and median overall survival up to 20.9 months in selected populations. Treatment-related grade ≥ 3 adverse events range from 10% to 25%, comparable to monotherapy approaches. However, outcomes vary across heterogeneous patient populations, and cross-trial comparisons remain limited. Ongoing prospective trials are evaluating combination strategies incorporating contemporary first-line regimens, including atezolizumab plus bevacizumab and the STRIDE regimen. **Conclusions**: Combination Y-90 radioembolization and immunotherapy demonstrates a strong biological rationale and encouraging early clinical signals, with acceptable safety profiles. However, current evidence remains preliminary and derived from non-randomized studies. Ongoing randomized trials are required to define optimal patient selection, treatment timing, and sequencing, and to establish whether combination therapy provides meaningful benefit over current standards of care.

## Simple Summary

Hepatocellular carcinoma (HCC) is frequently diagnosed at an advanced stage, limiting curative options. Immunotherapy has improved systemic disease control, while yttrium-90 (Y-90) radioembolization provides effective local tumor treatment. However, each modality has inherent limitations, including incomplete response rates and intrahepatic or extrahepatic disease progression. This review evaluates the rationale and emerging evidence supporting the combination of Y-90 radioembolization with immunotherapy. We summarize clinical outcomes, safety data, treatment sequencing considerations, and ongoing trials. Early data suggest potential synergistic effects, although optimal patient selection and timing remain to be defined.

## 1. Introduction

Hepatocellular carcinoma (HCC) is the most common primary liver malignancy and remains a leading cause of cancer-related mortality worldwide [1]. Despite advances in surveillance, many patients are diagnosed at an advanced stage, precluding curative options such as resection or transplantation. The median 5-year survival for HCC is 18–20%, which drops significantly to 2–7% in advanced, unresectable HCC [2,3,4,5].

Management of unresectable HCC requires a multidisciplinary approach integrating systemic therapy and liver-directed treatments. Immune checkpoint inhibitors (ICIs), particularly in combination with anti-angiogenic agents, have reshaped first-line systemic therapy. In parallel, Y-90 transarterial radioembolization (TARE) is an established locoregional therapy that provides durable local tumor control in appropriately selected patients [6,7].

Given the complementary mechanisms of systemic immune activation and localized tumor cytotoxicity, combination strategies have emerged as a promising therapeutic approach. This review synthesizes current evidence on Y-90 radioembolization combined with immunotherapy, focusing on biological rationale, clinical outcomes, safety, and ongoing trials, while highlighting limitations and areas of uncertainty.

## 2. Materials and Methods

This review used a semi-systematic (PRISMA-informed) approach to identify and synthesize evidence on immunotherapy, yttrium-90 (Y-90) radioembolization, and their combination for the treatment of hepatocellular carcinoma (HCC).

### 2.1. Search Strategy

A comprehensive literature search was performed in PubMed/MEDLINE for studies published through September 2025. Search terms included combinations of keywords and Medical Subject Headings (MeSH): “hepatocellular carcinoma,” “yttrium-90,” “radioembolization,” “transarterial radioembolization,” “immunotherapy,” “immune checkpoint inhibitors,” “PD-1,” “PD-L1,” “CTLA-4,” “VEGF,” and “biomarkers,” as well as specific agents including atezolizumab, durvalumab, tremelimumab, nivolumab, and pembrolizumab. Reference lists of relevant articles and review publications were manually screened to identify additional eligible studies.

The full PubMed search string used was as follows: (“Carcinoma, Hepatocellular” [Mesh] OR “hepatocellular carcinoma” [tiab] OR “hepatocellular carcinomas” [tiab] OR “liver cell carcinoma” [tiab] OR “HCC” [tiab]) AND (“radioembolization” [tiab] OR “radio-embolization” [tiab] OR “yttrium-90” [tiab] OR “Y-90” [tiab] OR “90Y” [tiab] OR “transarterial radioembolization” [tiab] OR “trans-arterial radioembolization” [tiab] OR “TARE” [tiab] OR “selective internal radiation therapy” [tiab] OR “SIRT” [tiab]) AND (“Immune Checkpoint Inhibitors” [Mesh] OR “immune checkpoint inhibitor” [tiab] OR “immune checkpoint inhibitors” [tiab] OR “checkpoint inhibitor” [tiab] OR “checkpoint inhibitors” [tiab] OR “immunotherapy” [tiab] OR “PD-1” [tiab] OR “PD-L1” [tiab] OR “CTLA-4” [tiab] OR “nivolumab” [tiab] OR “pembrolizumab” [tiab] OR “atezolizumab” [tiab] OR “durvalumab” [tiab] OR “tremelimumab” [tiab]). No language restrictions were applied.

### 2.2. Study Selection

Studies were included if they met the following criteria: (1) involved adult patients (≥18 years) with HCC; (2) evaluated Y-90 radioembolization, ICI-based immunotherapy, or their combination; and (3) reported clinical outcomes or relevant mechanistic findings. Eligible study designs included phase I–III clinical trials, prospective and retrospective cohort studies, real-world database analyses, and translational or mechanistic studies assessing tumor microenvironment or biomarker changes.

Case reports, editorials, and conference abstracts without full manuscripts were excluded, particularly for evaluation of combination therapy outcomes. Studies evaluating Y-90 radioembolization combined with non-immunotherapy systemic agents were reviewed for contextual purposes but were not the primary focus.

### 2.3. Data Extraction and Outcomes

**Figure 1:**
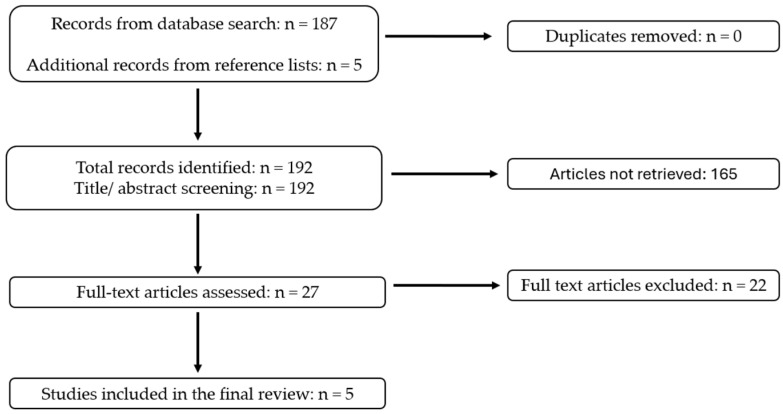
Literature search and study selection. Flow diagram demonstrating the database and reference-list records identified, screened, and assessed for eligibility.

Data were extracted on study design, patient population, treatment regimen, and reported outcomes. Clinical endpoints of interest included overall survival (OS), progression-free survival (PFS), time to progression (TTP), objective response rate (ORR), and treatment-related adverse events. Mechanistic studies were reviewed for evidence of immunomodulatory effects, including antigen release, immune cell activation, and pathway signaling. Four completed clinical studies were included in the final review. Ongoing trials were discussed separately to contextualize future directions [Figure 1]. Additional articles focused on HCC, systemic therapies, and Y-90 monotherapy, while the mechanistic rationale and general context are not included in the study selection flow diagram.

### 2.4. Data Synthesis

Given the heterogeneity in study design, patient populations, and treatment protocols, quantitative pooling was not performed. Findings were synthesized descriptively, with emphasis on the consistency of outcomes across studies and on the identification of emerging trends.

### 2.5. Methodological Considerations

To minimize selection bias, broad inclusion criteria were applied, and reference lists were systematically screened; however, given the narrative synthesis and predominance of early-phase and non-randomized studies, the findings should be interpreted as hypothesis-generating rather than definitive. The characterization of this review as PRISMA-informed reflects the structured literature search and explicit eligibility criteria, while still recognizing that it is narrative in nature rather than meta-analytic.

## 3. Systemic Therapy and Immunotherapy

Historically, systemic therapy for advanced hepatocellular carcinoma (HCC) was dominated by tyrosine kinase inhibitors (TKIs), including sorafenib and lenvatinib, which provided modest improvements in overall survival (OS). Over the past decade, however, immune checkpoint inhibitors (ICIs) have transformed the therapeutic landscape and are now central to first- and subsequent-line treatment strategies.

ICIs target key inhibitory pathways that regulate T-cell activation and tumor immune evasion. These include programmed cell death protein 1 (PD-1) (e.g., nivolumab, pembrolizumab), programmed death-ligand 1 (PD-L1) (e.g., atezolizumab, durvalumab), and cytotoxic T-lymphocyte–associated protein 4 (CTLA-4) (e.g., tremelimumab). PD-1/PD-L1 blockade disrupts inhibitory signaling between tumor cells and T lymphocytes, restoring cytotoxic T-cell activity within the tumor microenvironment. In parallel, CTLA-4 inhibition enhances early T-cell priming and proliferation, promoting expansion of effector T cells and facilitating tumor infiltration. Collectively, these mechanisms enhance antitumor immune responses and shift the tumor microenvironment toward an immune-active state.

In addition to immune checkpoint blockade, anti-angiogenic therapy plays a critical synergistic role. Vascular endothelial growth factor (VEGF) inhibition, most commonly with bevacizumab, not only suppresses tumor angiogenesis but also modulates the tumor microenvironment by improving immune cell infiltration and reducing immunosuppressive signaling. As such, combination regimens integrating ICIs with anti-VEGF therapy have become a cornerstone of modern HCC management.

This paradigm shift was established by the IMbrave150 phase III trial, which demonstrated that atezolizumab plus bevacizumab significantly improved survival compared with sorafenib, with a median OS of 19.2 months versus 13.4 months, respectively [8]. Similarly, the HIMALAYA trial demonstrated improved survival with the STRIDE regimen (single priming dose of tremelimumab combined with durvalumab maintenance), achieving a median OS of 16.4 months compared with 13.8 months in the sorafenib arm [9]. These regimens are now widely adopted as first-line standards of care in patients with preserved liver function.

Despite these advances, a substantial proportion of patients either do not respond to first-line immunotherapy or eventually develop disease progression. In this setting, second-line treatment options—including alternative ICIs, TKIs, and combination strategies—are increasingly utilized. Treatment selection requires a multidisciplinary approach and is guided by prior therapy exposure, liver function, tumor burden, and patient-specific factors. This evolving landscape underscores the need for novel combination strategies, including integration with locoregional therapies such as Y-90 radioembolization, to further improve outcomes.

## 4. Yttrium-90 Transarterial Radioembolization

Yttrium-90 (Y-90) microsphere therapy is a form of transarterial radioembolization (TARE). It represents an established locoregional treatment option for patients with hepatocellular carcinoma (HCC) across Barcelona Clinic Liver Cancer (BCLC) stages A–C. This technique involves the selective intra-arterial delivery of beta-emitting microspheres to tumor-feeding vasculature, enabling high-dose radiation to be delivered directly to tumor tissue while relatively sparing the surrounding hepatic parenchyma. Therapeutic goals include durable local tumor control, downstaging to resection or transplantation, and, in selected cases, curative intent.

An important distinction in Y-90 treatment is the types of microspheres available—glass and resin. Glass microspheres contain 2500 Bq per sphere. In contrast, resin microspheres contain 50 Bq per sphere, resulting in approximately a 50-fold difference in specific radiation activity. As a result, microscopic differences in dose distribution occur, but with appropriate dosing, comparable clinical outcomes can be seen. Typically, this means fewer glass microspheres need to be delivered to achieve comparable activity compared to resin microspheres. This difference also results in a more heterogeneous dose distribution with glass microspheres, due to the smaller number of spheres delivered. In comparison, a higher number of resin microspheres leads to a more homogeneous distribution.

Prospective studies, including the LEGACY and RASER trials, have demonstrated high objective response rates and durable disease control in patients with solitary or limited HCC, supporting the role of Y-90 in carefully selected early-stage disease [6,10]. Across broader patient populations, reported median overall survival (OS) following Y-90 radioembolization ranges from approximately 6 to 24 months, with outcomes influenced by tumor burden, liver function, BCLC stage, and the presence of vascular invasion [11,12,13].

Advances in personalized dosimetry and radiation segmentectomy have further refined this approach, enabling delivery of ablative radiation doses to target liver segments. In selected patients, these strategies have been associated with near-curative outcomes, including objective response rates approaching 70% and meaningful improvements in survival [14].

Comparative studies have also evaluated Y-90 relative to other arterial therapies. In the TRACE trial, Y-90 radioembolization was associated with improved overall survival compared with transarterial chemoembolization (TACE) (30.2 vs. 15.6 months), highlighting its potential advantage in selected patient populations [15]. However, such findings should be interpreted in the context of patient selection and trial design.

Within the current treatment landscape, Y-90 radioembolization is most commonly utilized in patients with liver-limited disease and preserved hepatic function, particularly in early- and intermediate-stage HCC. Treatment planning incorporating personalized dosimetry has become increasingly important to optimize therapeutic efficacy while minimizing toxicity.

## 5. Rationale Supporting Combination Therapy

Despite advances in both immunotherapy and yttrium-90 (Y-90) radioembolization, each modality has inherent limitations. As with other locoregional therapies, Y-90 provides durable local tumor control but does not adequately address intrahepatic or extrahepatic disease progression. Recurrence outside the treated radiation field remains common, with up to 62% of patients developing new lesions within two years following treatment despite effective control of targeted tumors [16]. Additionally, the ability to deliver uniform ablative radiation doses—estimated to be on the order of 400 Gy for complete tumor necrosis—may be limited in practice by safety constraints and tumor heterogeneity [6].

Conversely, although immune checkpoint inhibitors (ICIs) have improved systemic therapy outcomes, objective response rates remain modest, typically ranging from approximately 11% to 36%, with disease control rates of 55% to 74% across regimens [17,18]. A substantial proportion of patients, therefore, fail to achieve durable responses with immunotherapy alone. Taken together, these complementary limitations provide a strong rationale for combining locoregional and systemic treatment approaches to enhance overall therapeutic efficacy.

The biological rationale for combining Y-90 radioembolization with immunotherapy is supported by a growing body of translational evidence demonstrating the immunomodulatory effects of radiation. In addition to direct cytotoxicity, radiation induces immunogenic cell death, resulting in the release of tumor-associated antigens, neoantigens, and proinflammatory cytokines. These processes enhance antigen presentation, promote dendritic cell activation, and facilitate recruitment and infiltration of tumor-specific cytotoxic T lymphocytes into the tumor microenvironment [19,20]. Consistent with this, increased expression of genes involved in both innate and adaptive immune responses has been observed following Y-90 treatment [21].

Radiation-induced immune activation also exhibits a dynamic temporal profile. Early reductions in interferon (IFN) signaling, particularly IFN-γ, may be followed by delayed upregulation several weeks after treatment, suggesting a sustained and evolving immune response that extends beyond the period of radiation delivery. This delayed immune activation may contribute to the prolonged antitumor effects observed after Y-90 radioembolization.

At the molecular level, Y-90–induced DNA damage activates the stimulator of interferon genes (STING) pathway, leading to increased type I interferon production. This signaling cascade promotes dendritic cell maturation, enhances cross-presentation of tumor antigens, and facilitates CD8+ T-cell priming, thereby amplifying systemic antitumor immunity [21,22]. While these mechanisms have been hypothesized to contribute to the abscopal effect—tumor regression at sites distant from the radiation field—clinical evidence supporting consistent abscopal responses following Y-90 alone remains limited [23].

Rather than relying on abscopal effects, the combination of Y-90 radioembolization and immunotherapy is more plausibly explained by complementary and potentially synergistic mechanisms. Radiation may enhance tumor immunogenicity and increase susceptibility to immune checkpoint blockade, thereby converting immunologically “cold” tumors into “hot,” inflamed tumors more responsive to ICIs. This mechanistic rationale for synergy provides a strong foundation for ongoing clinical investigation of combination strategies.

These microscopic differences may have significant implications for the nature and intensity of radiation-induced “conversion” of the tumor immune environment. High-dose focal radiation with glass microspheres may lead to a concentrated area of STING pathway activation, with more robust interferon production. On the other hand, resin microspheres may convert a larger volume of the tumor microenvironment to an immunologically “hot” area, but with less concentrated/intense immune reactions. These differences in immune response and microsphere type are not characterized in the current literature. Future studies that stratify biomarker response by microsphere type are needed to assess differences in remodeling of the microenvironment when combined with immunotherapy and whether those differences are clinically meaningful.

**Figure 2:**
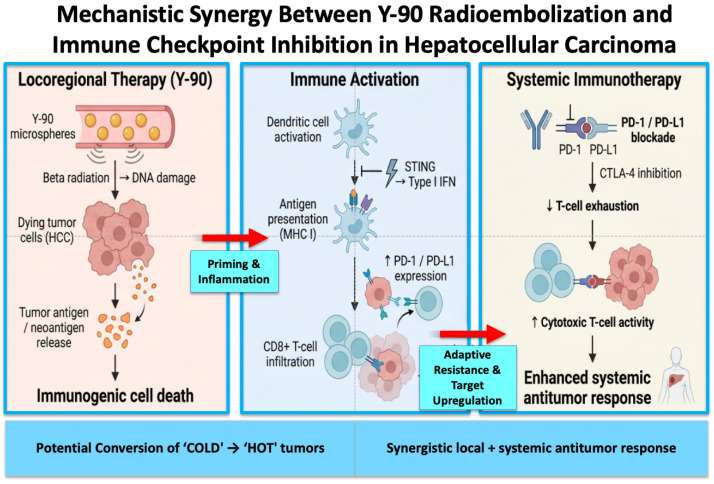
Mechanistic synergy between Y-90 radioembolization and immune checkpoint inhibition in hepatocellular carcinoma. Y-90 radioembolization induces tumor cell death and DNA damage, resulting in the release of tumor-associated antigens and neoantigens. This promotes dendritic cell activation, antigen presentation, and recruitment of tumor-infiltrating lymphocytes, accompanied by activation of the STING pathway and interferon signaling. Concurrent upregulation of PD-1/PD-L1 pathways may contribute to T-cell exhaustion. Immune checkpoint inhibitors restore cytotoxic T-cell function by blocking inhibitory signaling, thereby enhancing systemic antitumor immunity. Together, these processes may convert immunologically “cold” tumors into “hot” tumors, thereby improving local and systemic tumor control.

Collectively, these translational findings support the hypothesis that Y-90 radioembolization may act as an in situ immunologic primer, sensitizing tumors to immune checkpoint inhibition (Figure 2). This concept has gained early clinical support, with emerging studies demonstrating encouraging activity of combination approaches, although definitive benefit remains to be established in randomized settings. It is important to note that true biological synergy has not been demonstrated in the current literature. Translational and randomized controlled trials that rigorously evaluate this mechanism are needed to establish it.

## 6. Current Clinical Evidence for Immunotherapy and Y-90 Radioembolization

### 6.1. Early Retrospective and Phase I/II Prospective Clinical Studies

Early retrospective and prospective studies provide preliminary evidence supporting the feasibility and safety of combining immunotherapy with Y-90 radioembolization, with signals of clinical activity observed across multiple cohorts. However, interpretation of these findings is limited by small sample sizes, heterogeneous patient populations, and predominantly non-randomized study designs (Table 1).

One of the earliest reports was a single-center retrospective study of 26 patients who received immunotherapy within 90 days of Y-90 radioembolization [24]. The majority of patients had preserved performance status (ECOG 0–1), and 81% had advanced disease (BCLC stage C). The combination approach was generally well tolerated, with only two patients experiencing delayed grade 3–4 toxicities. Median overall survival (OS) was 16.5 months, which appears comparable to historical outcomes reported for Y-90 or immunotherapy alone. Prior studies have reported median OS of approximately 11.3 months for Y-90 in BCLC stage C disease and 15–19 months for immunotherapy alone [25,26,27]. However, cross-study comparisons should be interpreted cautiously, given differences in patient selection and treatment heterogeneity.

Building on these findings, early-phase prospective studies have further explored the combination of Y-90 radioembolization followed by immune checkpoint inhibitors (ICIs). In a phase II study from Singapore, Tai et al. evaluated Y-90 followed by nivolumab in 36 patients with advanced HCC [28]. Median OS was 12.9 months, with an objective response rate (ORR) of 30.6% and median progression-free survival (PFS) of 3.6 months. Grade ≥ 3 adverse events occurred in 14% of patients. Although these results demonstrated an acceptable safety profile, response rates did not clearly exceed those reported with contemporary first-line systemic therapies.

Importantly, this study also provided translational insight into tumor immune dynamics. Paired tumor biopsies demonstrated that responders exhibited conversion from immunologically “cold” to “hot” or mixed tumor phenotypes following treatment, characterized by increased interferon-γ–related signaling, mutation burden, and macrophage activity. However, pre-treatment immune phenotype did not reliably predict response, highlighting the current limitations of biomarker-driven patient selection.

A subsequent multicenter phase II study evaluated Y-90 followed by nivolumab in a more homogeneous cohort of patients with BCLC stage B2 or C disease and segmental or lobar portal vein invasion [29]. Among 41 treated patients, the ORR was 41.5%, with a median time to progression (TTP) of 8.8 months, a median PFS of 9.0 months, and a median OS of 20.9 months. These outcomes appear favorable relative to historical controls in similar patient populations, in which PFS typically ranges from 3 to 6 months and median OS from 8 to 19 months [30,31,32,33]. Nevertheless, the absence of a control arm and the selection of patients ineligible for transarterial chemoembolization (TACE) limit definitive conclusions regarding comparative efficacy. Treatment-related toxicity was manageable, with grade 3–4 adverse events reported in 25% of patients, without evidence of synergistic toxicity between modalities.

Additional prospective data have been reported with other ICIs. In a phase I/II study evaluating Y-90 radioembolization followed by durvalumab in 24 patients with BCLC stage B or C disease (Child–Pugh score < 7), median TTP was 15.2 months, and median PFS was 6.9 months [34]. Fewer than 10% of patients experienced grade ≥ 3 adverse events, supporting an acceptable safety profile. When considered in the context of contemporary systemic therapy, these outcomes appear comparable to those observed with atezolizumab plus bevacizumab, which demonstrates a median PFS of approximately 6–7 months in similar populations [8,17]. However, differences in study design, patient selection, and endpoints preclude direct comparison.

Collectively, early-phase studies suggest that combining Y-90 radioembolization with immunotherapy is feasible and generally well tolerated, with signals of antitumor activity in selected patient populations. However, the current evidence remains preliminary, and findings should be interpreted as hypothesis-generating. Ongoing randomized and prospective studies will be essential to define the true clinical benefit, optimal patient selection, and appropriate sequencing of these therapies.

**Table 1:** Early clinical studies combining Y-90 radioembolization and immunotherapy in HCC treatment.

Study	Design	N	Population	Microsphere Type	Treatment Regimen	Response Criteria	ORR (%)	Median OS (mo)	Median PFS/TTP (mo)	Grade ≥ 3 AE (%)	Key Limitation
Zhan et al. [24]	Retrospective	26	CP A–B7; mostly BCLC C	23 patients—Glass 3 patients—Resin	ICI within 90 days of Y-90	Hepatic tumors: mRECIST criteria Extrahepatic disease: RECIST criteria	NR	16.5	PFS 5.7	Low (2 pts)	Small, heterogeneous
Tai et al. [28]	Phase II	36	Advanced HCC	Resin	Y-90 → nivolumab	RECIST criteria	30.6	12.9	PFS 3.6	14%	No comparator
De La Torre-Alaez et al. [29]	Phase II multicenter	41	BCLC B2/C (PVI)	Resin	Y-90 → nivolumab	RECIST criteria	41.5	20.9	PFS 9.0/TTP 8.8	25%	No control arm
Lee et al. [34]	Phase I/II	24	CP < 7, BCLC B/C	Glass	Y-90 → durvalumab	mRECIST	83.3	NR	PFS 6.9/TTP 15.2	<10%	Small sample size

Abbreviations: BCLC, Barcelona Clinic Liver Cancer; CP, Child—Pugh; ORR, objective response rate; OS, overall survival; PFS, progression-free survival; TTP, time to progression; PVI, portal vein invasion.

### 6.2. Ongoing Phase II/III Prospective Studies

Ongoing prospective studies are expected to play a critical role in defining the clinical utility of combining immunotherapy with Y-90 radioembolization, including optimal patient selection, treatment sequencing, and comparative efficacy relative to established standards of care.

The EMERALD-Y90 study is an ongoing phase II single-arm trial evaluating the safety and efficacy of Y-90 radioembolization followed by systemic immunotherapy in patients with unresectable HCC or those who have declined curative therapies. Following Y-90 treatment, patients receive an initial dose of durvalumab, followed by a combination of durvalumab and bevacizumab administered every three weeks. Key inclusion criteria include preserved liver function (Child–Pugh class A), ECOG performance status 0–1, and an adequate functional liver remnant (>30%). Patients with major portal vein invasion, extrahepatic disease, or prior systemic therapy are excluded. This study aims to evaluate whether sequential integration of locoregional and systemic therapy can enhance clinical outcomes in a carefully selected population.

Another ongoing phase II study, the ROWAN trial, is investigating the combination of Y-90 radioembolization with the STRIDE regimen. In this protocol, patients who are not candidates for, or decline, surgical intervention undergo Y-90 treatment with glass microspheres, followed by a single priming dose of tremelimumab and maintenance durvalumab every 4 weeks. The rationale for this approach is supported by the HIMALAYA trial, which demonstrated improved survival and objective response rates with the STRIDE regimen compared with sorafenib in advanced HCC [9]. The ROWAN study will provide important insights into whether dual immune checkpoint blockade combined with locoregional therapy can further enhance antitumor activity.

In addition, a multicenter, open-label randomized phase II trial is evaluating Y-90 radioembolization in combination with atezolizumab and bevacizumab in patients with unresectable intermediate-stage HCC [35]. Eligible patients include those with BCLC stage B disease who are not candidates for downstaging strategies. Participants are randomized to receive Y-90 radioembolization alone or in combination with atezolizumab plus bevacizumab, enabling direct comparison of combination therapy versus locoregional treatment alone. This study is particularly important, as it addresses the incremental benefit of adding systemic immunotherapy to Y-90 within a contemporary treatment framework.

Collectively, these ongoing trials represent a critical step toward establishing the role of Y-90 radioembolization combined with immunotherapy in HCC management. However, until results from randomized and adequately powered studies become available, the clinical benefit of combination therapy remains to be definitively established. Despite encouraging early-phase data, no randomized study has yet demonstrated the superiority of combination therapy over current first-line systemic regimens, representing a critical evidence gap.

## 7. Clinical Positioning and Implications

Within the current treatment paradigm, immune checkpoint inhibitor–based combinations, particularly atezolizumab plus bevacizumab and the STRIDE regimen, represent the standard first-line systemic therapy for patients with advanced hepatocellular carcinoma and preserved liver function [8,9]. Y-90 radioembolization, in contrast, is most commonly utilized in patients with liver-limited disease, particularly in early- and intermediate-stage HCC, where it provides effective local tumor control. The integration of these approaches therefore represents a rational strategy to bridge locoregional and systemic disease control.

At present, the role of combining Y-90 radioembolization with immunotherapy remains investigational. Ongoing phase II and III prospective studies are evaluating key clinical endpoints, including progression-free survival and overall survival, in patients with unresectable and locally advanced HCC. Notably, current trials primarily enroll patients with preserved liver function (Child–Pugh class A), good performance status, and no extrahepatic disease, reflecting a relatively selected population. If these studies demonstrate definitive benefit, they will be instrumental in refining patient selection and clarifying the optimal role of combination therapy within existing treatment algorithms.

Potential clinical applications include patients with liver-dominant disease at high risk of intrahepatic progression, those with suboptimal response to systemic therapy alone, or select intermediate-stage patients who are not optimal candidates for transarterial chemoembolization. However, important uncertainties remain, including optimal timing, sequencing, and treatment intensity. As such, further prospective investigation is required to define the most appropriate patient population and treatment strategy. Until robust randomized data are available, the use of combination therapy should be individualized and considered within multidisciplinary discussion or clinical trial settings.

Stratifying by BCLC stage, liver function, and tumor burden provides a more clinically instructive framework for patient selection. In BCLC stage A–B patients with Child–Pugh class A function and liver-limited disease, Y-90 radioembolization combined with immunotherapy may be most appropriate when TACE candidacy is limited or when downstaging to resection or transplantation is the therapeutic goal; in this population, preserved hepatic reserve best accommodates the cumulative hepatotoxicity risks of combination therapy. In patients with large or multifocal BCLC stage B disease and Child–Pugh class A function who are poor TACE candidates, the combination may offer particular value by coupling effective locoregional control with systemic immune activation targeting disease beyond the treatment field. In BCLC stage C patients with portal vein invasion or extrahepatic spread, the combination strategy is most logically positioned as a complement to first-line systemic therapy; here, Y-90 addresses dominant intrahepatic disease while immunotherapy targets systemic dissemination. Patients with Child–Pugh B7 disease are underrepresented in current prospective trials. At the same time, retrospective data suggest that Y-90 can be performed safely in selected individuals; however, the incremental hepatotoxicity of ICI therapy necessitates careful risk stratification. High bilateral tumor burden and reduced functional liver reserve may limit deliverable radiation dose and narrow the therapeutic window of combination approaches. These proposed clinical scenarios require prospective validation, and combination therapy should be applied within multidisciplinary teams and, where feasible, within the framework of clinical trials.

An emerging clinical application of Y-90 radioembolization combined with immunotherapy is its potential role in “conversion therapy”, defined as downstaging initially unresectable disease to enable curative-intent resection or liver transplantation. Patients with advanced HCC complicated by macrovascular portal venous invasion (PVI), including lobar or main portal vein tumor thrombus (PVTT), represent a particularly challenging subgroup for whom curative options have historically been limited. Early prospective data support the feasibility of Y-90 in this population; the phase II multicenter study by De La Torre-Alaez et al. enrolled patients exclusively with BCLC stage B2 or C disease and segmental or lobar PVI, reporting an objective response rate of 41.5% and a median overall survival of 20.9 months [29]—favorable relative to historical benchmarks or PVTT, in which median OS without effective locoregional therapy typically ranges from 2 to 8 months. The addition of immune checkpoint inhibitors to radioembolization in this setting may further augment tumor response and expand the proportion of patients who can be down-staged, potentially repositioning the combination as a bridge to curative therapy in select patients with macrovascular invasion.

A related clinical question is how Y-90 radioembolization plus immunotherapy compares with alternative locoregional strategies in advanced HCC and PVTT. Conventional TACE is generally contraindicated in patients with main portal vein invasion due to the risk of hepatic ischemia; Y-90 radioembolization can typically be administered more safely in this population, given its less embolic mechanism. Hepatic arterial infusion chemotherapy (HAIC), particularly FOLFOX-HAIC, has demonstrated high response rates (approximately 40–50%) in Asian cohorts with PVTT, and combinations with sorafenib or lenvatinib have shown encouraging activity; however, HAIC requires repeated arterial procedures and specialized expertise, limiting its broader adoption. Compared with TACE plus anti-PD-1/PD-L1 therapy, which has been evaluated in several Asian trials with favorable response data, Y-90 radioembolization may offer a more immunogenic treatment platform by virtue of its radiation-induced immunogenic cell death and STING pathway activation. Whether this translates into superior clinical outcomes when combined with immunotherapy remains to be established, as direct comparative data between Y-90-plus-ICI and alternative locoregional-systemic strategies are currently lacking. Prospective head-to-head studies are required to define the relative merits of these approaches in patients with advanced HCC and macrovascular invasion.

## 8. Immunotherapy and Anti-Angiogenic Agents

A range of immune checkpoint inhibitors (ICIs) has been investigated in combination with Y-90 radioembolization, reflecting the expanding role of immunotherapy in hepatocellular carcinoma (HCC). Early clinical studies have evaluated agents targeting the programmed cell death protein 1 (PD-1) pathway (e.g., nivolumab, pembrolizumab), the programmed death-ligand 1 (PD-L1) pathway (e.g., durvalumab, atezolizumab), and the cytotoxic T-lymphocyte–associated protein 4 (CTLA-4) pathway (e.g., tremelimumab). However, no head-to-head comparative studies have been performed, and the optimal immunotherapeutic partner for Y-90 radioembolization remains undefined.

Mechanistically, PD-1/PD-L1 blockade may be particularly relevant in the post-radiation setting. Following Y-90 treatment, increased antigen release and immune activation can lead to upregulation of inhibitory checkpoint pathways, contributing to T-cell exhaustion. In this context, PD-1/PD-L1 inhibitors may restore cytotoxic T-cell function and sustain antitumor immune responses. In contrast, CTLA-4 inhibition—central to regimens such as STRIDE—acts earlier in the immune response by enhancing T-cell priming, expansion, and tumor infiltration. These complementary mechanisms suggest that different classes of ICIs may have distinct, and potentially synergistic, roles when combined with locoregional therapy.

Ongoing prospective trials incorporating contemporary first-line regimens, including atezolizumab plus bevacizumab and the STRIDE regimen, are expected to provide further insight into the optimal immunotherapeutic backbone for combination strategies. However, larger randomized studies will be required to determine whether specific ICIs confer superior efficacy when paired with Y-90 radioembolization.

In addition to immune checkpoint blockade, anti-angiogenic therapy represents an important adjunct in this setting. Y-90 radioembolization has been associated with transient upregulation of angiogenic factors, including vascular endothelial growth factor (VEGF), which may contribute to tumor progression following treatment [36,37]. In a pilot study evaluating post-treatment angiogenic signaling, elevated VEGF levels were observed in patients with poorer overall survival (<6 months), suggesting a potential association between angiogenic rebound and adverse outcomes [38]. The addition of anti-VEGF therapy may therefore enhance treatment efficacy by mitigating radiation-induced angiogenic signaling while simultaneously improving immune cell infiltration and modulating the tumor microenvironment.

Collectively, these observations support a multifaceted strategy in which Y-90 radioembolization, immune checkpoint inhibition, and anti-angiogenic therapy may act through complementary pathways. Nonetheless, the optimal combination regimen and sequencing strategy remain to be defined and require validation in prospective clinical trials.

When comparing the mechanistic contributions of anti-VEGF therapy and CTLA-4 inhibition as synergistic partners for Y-90 radioembolization, several distinctions are relevant. Anti-VEGF therapy, as provided by bevacizumab, may be particularly well-suited to the immediate post-radioembolization period: Y-90 induces a transient angiogenic rebound characterized by elevated serum VEGF [36,37,38], which may be mitigated by concurrent VEGF blockade, and bevacizumab simultaneously normalizes aberrant tumor vasculature, thereby improving immune effector cell infiltration. These effects may amplify the immune priming initiated by radiation-induced antigen release. In this context, atezolizumab plus bevacizumab represents a mechanistically coherent triplet approach when combined with Y-90. CTLA-4 inhibition, as embodied in tremelimumab within the STRIDE regimen, primarily operates during the priming phase of adaptive immunity, enhancing T-cell expansion and tumor infiltration. Y-90 radioembolization, by increasing neoantigen presentation and dendritic cell activation, may amplify the T-cell priming facilitated by CTLA-4 blockade, a complementary mechanism that operates over a longer temporal window. Post-radiation upregulation of PD-L1 within the tumor microenvironment provides an additional rationale for PD-1/PD-L1 blockade to sustain downstream effector T-cell function. On this basis, the authors hypothesize that anti-VEGF–based combinations may offer more proximate synergy in the early post-radiation period. Meanwhile, CTLA-4 inhibition may more effectively leverage the downstream adaptive immune consequences of radiation. These mechanisms are not mutually exclusive; the dual checkpoint inhibition of the STRIDE regimen may synergize with both early and late radiation-induced immune effects. This mechanistic hierarchy nonetheless remains unvalidated, and prospective trials will be required to determine whether anti-VEGF or CTLA-4 inhibition offers superior synergistic benefit as part of a Y-90-based combination regimen.

## 9. Timing of Y-90 Radioembolization and Immunotherapy

The optimal timing of Y-90 radioembolization in relation to immunotherapy remains an important but unresolved question. Following Y-90 treatment, an acute inflammatory response occurs, and early initiation of immune checkpoint inhibitors (ICIs) may increase the risk of overlapping toxicities, including hepatic dysfunction and transaminitis. ICIs are also associated with immune-related adverse events, such as immune-mediated hepatitis, dermatitis, and endocrinopathies. In addition, transient lymphopenia following radioembolization may further modulate immune responses and influence treatment tolerance. Despite these considerations, early-phase studies have demonstrated that short intervals between therapies—on the order of 7–14 days—are feasible and associated with acceptable safety profiles [34].

Conversely, excessive delay in initiating immunotherapy may attenuate the potential for therapeutic synergy. Radiation-induced immune activation is temporally dynamic, with evidence of PD-1 upregulation following Y-90 radioembolization [21]. As ICIs directly target this pathway, early initiation may enhance treatment efficacy by capitalizing on this window of immune activation. However, prolonged PD-1 upregulation has been associated with T-cell exhaustion and poorer clinical outcomes, suggesting that delayed treatment may be less effective [39]. Collectively, these observations support the concept of a potentially narrow therapeutic window, in which immunotherapy should ideally be initiated during peak immune activation but prior to the development of immune exhaustion. This temporal framework also highlights the potential role of biomarkers in guiding treatment timing and patient selection.

Based on the available mechanistic and early clinical evidence, the authors propose the following hypothetical treatment timeline as a clinically instructive framework, while acknowledging that it remains provisional pending prospective validation. Following Y-90 radioembolization, an interval of approximately 1–2 weeks is recommended to allow resolution of the acute inflammatory response and stabilization of hepatic function, consistent with the feasibility demonstrated in early-phase studies utilizing short therapy intervals [34]. Initiation of ICI therapy at approximately 2–4 weeks post-Y-90 is proposed as the optimal window for therapeutic synergy, coinciding with the period of maximal radiation-induced antigen release, dendritic cell activation, and early STING-mediated interferon signaling. Initiating PD-1/PD-L1 blockade within this window is expected to prevent premature T-cell exhaustion associated with sustained PD-1 upregulation, while avoiding compounded toxicity from immediate post-procedural immune activation. For regimens incorporating CTLA-4 inhibition (e.g., the STRIDE regimen), the priming dose could theoretically be administered concurrently with or shortly after this window, leveraging peak antigen presentation to maximize T-cell expansion. Delaying ICI initiation beyond 6–8 weeks post-Y-90 is likely suboptimal from a synergy standpoint; however, delayed initiation may be clinically necessary in patients with delayed hepatic recovery or persistent transaminase elevation. Serial monitoring of hepatic function (AST, ALT, bilirubin) during this transition period remains essential to distinguish radiation-embolization-induced liver disease from immune-mediated hepatitis and to guide treatment decisions. This proposed timeline represents the authors’ expert interpretation of current mechanistic and clinical data. It should be understood as a hypothesis-generating framework to inform clinical trial design, rather than an established clinical standard.

## 10. Predictors of Response

Identifying predictors of response remains a critical step in optimizing combination strategies. Tumor-intrinsic immune characteristics, including the distinction between immunologically “hot” and “cold” tumors, have been proposed as potential biomarkers of response to immunotherapy. However, existing data remain inconsistent. While some studies suggest that inflamed tumor microenvironments are associated with improved outcomes, the study by Tai et al. did not demonstrate a reliable association between pre-treatment immune phenotype and treatment response [28].

These findings underscore the complexity of tumor–immune interactions in HCC and suggest that static biomarkers alone may be insufficient to predict response. Dynamic changes in the tumor microenvironment following Y-90 radioembolization—including antigen release, immune cell infiltration, and cytokine signaling—may be more relevant determinants of treatment efficacy. As such, further investigation into integrated biomarker strategies, including molecular, immunologic, and imaging-based approaches, will be essential. Ongoing studies are expected to clarify the predictive value of these factors and to better define patient subgroups most likely to benefit from combination therapy. A challenge facing the current literature is the lack of translational research from preclinical mechanistic evidence and human clinical data to support combination therapy. The current biological rationale focuses on either murine models of radiation-induced immunologic cell death or STING pathway activation. In humans with HCC, this may not completely capture the disease state that commonly arises from chronic liver inflammation and fibrosis. The study by Tai et al. demonstrates the limits of current human translational research, including the technical challenges of obtaining pre- and post-treatment biopsies, which result in small biopsy cohorts.

Future translational clinical studies assessing pre- and post-Y90 treatment, which include flow cytometry and RNA sequencing, will assist in validating preclinical mechanisms. Currently, the mechanism of combination therapy with Y-90 and immunotherapy is a hypothesis that requires thorough translational studies to provide a better mechanistic understanding.

## 11. Long-Term Safety Profile

Available clinical data suggest that the combination of Y-90 radioembolization and immunotherapy is generally well tolerated, with a safety profile comparable to that observed with each modality alone. However, follow-up in most studies remains limited, typically not exceeding 24 months, with only a few reports extending beyond approximately three years. As such, the long-term impact of combination therapy on hepatic function and overall patient outcomes remains incompletely characterized.

One well-documented risk of using bevacizumab in patients with HCC and underlying cirrhosis and portal hypertension is the risk of bleeding. VEGF inhibition can jeopardize hemeostasis, which can have significant complications for patients with esophageal or gastric varices. This risk has resulted in an FDA boxed warning, requiring careful patient selection and monitoring when treating with bevacizumab. In the Imbrave150 trial, enrolled patients underwent variceal screening and, if necessary, management.

Importantly, most prospective studies have enrolled patients with preserved liver function (Child–Pugh class A), whereas in clinical practice, patients with more advanced liver dysfunction are frequently considered for treatment. Retrospective studies suggest that Y-90 radioembolization can be performed in selected patients with Child–Pugh class B7 disease, although the risk of hepatic decompensation is increased [40]. Similarly, retrospective analyses of ICIs in HCC suggest comparable safety profiles between Child–Pugh class A and B patients, although these findings require further validation [41].

One well-known complication of radioembolization is radiation-embolization-induced liver disease (REILD), which is often driven by radiation-induced sinusoidal obstruction. This often presents 4–8 weeks after Y-90 radioembolization due to sinusoidal obstruction, without bile duct obstruction or tumor growth. A similar but biologically distinct presentation can occur when Y-90 radioembolization is combined with ICIs, leading to immune-mediated hepatitis (IMH). In IMH, the histopathology demonstrates lobular hepatitis with CD8+ T-cell infiltration. Both disease processes can present with elevated LFTs, jaundice, and, in extreme cases, hepatic decompensation. IMH develops 4–12 weeks after ICI initiation, but can more often be asymptomatic and be detected incidentally. Patients who receive Y-90 radioembolization and ICI combination therapy can have adverse effects, including hepatotoxicity around 4–8 weeks. With overlapping timelines and clinical features, distinguishing REILD from IMH can be difficult. Typically, REILD results from sinusoidal endothelial cells becoming injured, leading to inflammation and coagulation. This vascular injury can lead to sinusoidal obstructive symptoms, such as jaundice and ascites. As a result, REILD can present with a more cholestatic pattern of hepatic injury, whereas a hepatocellular pattern can suggest IMH. Clinical studies investigating combination treatment should attempt to elucidate which hepatotoxicity patients are encountering, as treatments and risk factors differ.

As therapeutic advances extend survival and shift treatment earlier in the disease course, preservation of hepatic reserve will become increasingly important. With this, understanding the underlying mechanism of injury will also become crucial. While late toxicity may currently have a limited impact due to the overall prognosis of advanced HCC, this may change as outcomes improve. Accordingly, long-term safety, particularly regarding cumulative hepatic injury, is an important area for future investigation.

## 12. Conclusions

Early clinical and translational studies support a biologically plausible and potentially synergistic interaction between Y-90 radioembolization and immunotherapy in hepatocellular carcinoma. While preliminary data suggest acceptable safety and signals of clinical activity, the current evidence base remains limited by small, non-randomized studies and selected patient populations.

Key challenges remain, including defining optimal treatment timing, sequencing, and patient selection, as well as identifying reliable biomarkers of response. Ongoing prospective and randomized trials will be essential to determine whether combination therapy provides meaningful clinical benefit over established standards of care.

At present, the integration of Y-90 radioembolization and immunotherapy should be considered investigational. However, as understanding of tumor biology and immune dynamics continues to evolve, this strategy holds promise as a means of bridging locoregional and systemic therapy to improve outcomes in selected patients with HCC.

## Data Availability

No new data were created or analyzed in this study. Data sharing is not applicable to this article.

## Abbreviations

The following abbreviations are used in this manuscript:

AE/AEs	Adverse event(s)
BCLC	Barcelona Clinic Liver Cancer (staging system)
CP	Child-Pugh (liver function classification)
CTLA-4	Cytotoxic T-lymphocyte-associated protein 4
ECOG	Eastern Cooperative Oncology Group
HCC	Hepatocellular carcinoma
ICI/ICIs	Immune checkpoint inhibitors
IFN	Interferon
mRNA	Messenger ribonucleic acid
ORR	Objective response rate
OS	Overall survival
PD-1	Programmed cell death protein 1
PD-L1	Programmed cell death-ligand 1
PFS	Progression-free survival
PS	Performance status
PVI	Portal vein invasion
STING	Stimulator of interferon genes
TACE	Transarterial chemoembolization
TARE	Transarterial radioembolization
Th1	T helper type 1
TKI/TKIs	Tyrosine kinase inhibitor(s)
TTP	Time to progression
VEGF	Vascular endothelial growth factor
Y-90/Y90	Yttrium-90
